# Thermal and nutritional environments during development exert different effects on adult reproductive success in *Drosophila melanogaster*

**DOI:** 10.1002/ece3.7064

**Published:** 2020-11-24

**Authors:** Kyeong Woon Min, Taehwan Jang, Kwang Pum Lee

**Affiliations:** ^1^ Department of Agricultural Biotechnology Seoul National University Seoul Korea

**Keywords:** development, fecundity, fitness, life‐history trait, lifespan, macronutrients, silver spoon effects, temperature

## Abstract

Environments experienced during development have long‐lasting consequences for adult performance and fitness. The “environmental matching” hypothesis predicts that individuals perform best when adult and developmental environments match whereas the “silver spoon” hypothesis expects that fitness is higher in individuals developed under favorable environments regardless of adult environments. Temperature and nutrition are the two most influential determinants of environmental quality, but it remains to be elucidated which of these hypotheses better explains the long‐term effects of thermal and nutritional histories on adult fitness traits. Here we compared how the temperature and nutrition of larval environment would affect adult survivorship and reproductive success in the fruit fly, *Drosophila melanogaster*. The aspect of nutrition focused on in this study was the dietary protein‐to‐carbohydrate (P:C) ratio. The impact of low developmental and adult temperature was to improve adult survivorship. High P:C diet had a negative effect on adult survivorship when ingested during the adult stage, but had a positive effect when ingested during development. No matter whether adult and developmental environments matched or not, females raised in warm and protein‐enriched environments produced more eggs than those raised in cool and protein‐limiting environments, suggesting the presence of a significant silver spoon effect of larval temperature and nutrition. The effect of larval temperature on adult egg production was weak but persisted across the early adult stage whereas that of larval nutrition was initially strong but diminished rapidly after day 5 posteclosion. Egg production after day 5 was strongly influenced by the P:C ratio of the adult diet, indicating that the diet contributing mainly to reproduction had shifted from larval to adult diet. Our results highlight the importance of thermal and nutritional histories in shaping organismal performance and fitness and also demonstrate how the silver spoon effects of these aspects of environmental histories differ fundamentally in their nature, strength, and persistence.

## Graphical abstract

The effects of early environments on adult reproductive outcome were substantially influenced by thermal and nutritional histories experienced early in life. The nature and strength of these early environmental effects differed markedly depending on whether they were mediated by temperature or diet.

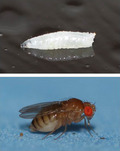

## 1 INTRODUCTION

Environments experienced during development not only exert direct effects on juvenile growth and development, but also have far‐reaching consequences for adult life‐history traits that have direct bearings on Darwinian fitness (Bateson et al., 2004; Gluckman & Hanson, 2004; Monaghan, 2008). Since the fitness of an organism is an integrated outcome of its past and present environments (Tab orsky, 2006), the complete understanding of the effects of environments on organismal fitness requires a detailed and accurate assessment of the interactions between developmental and adult environments. The long‐term fitness consequences of developmental environments have been mainly documented from vertebrates (Lindström, 1999; Lummaa & Clutton‐Brock, 2002), but there are also ample studies describing the significant impacts of developmental environments on adult fitness in insects (Barrett et al., 2009; Bauerfeind & Fischer, 2005; Boggs & Freeman, 2005; Dmitriew & Rowe, 2011; Duxbury & Chapman, 2020; Grangeteau et al., 2018; May et al., 2015; Runagall‐McNaull et al., 2015; Stefana et al., 2017).

There are two competing hypotheses formulated to explain how developmental environments interact with adult environments to shape the fitness of an individual organism. Firstly, the “environmental matching” or “predictive adaptive response” hypothesis posits that developing individuals can adaptively adjust their physiology and developmental trajectories so as to fit into an adult environment that is predicted by the environment experienced during development (Bateson et al., 2004; Gluckman et al., 2005; Monaghan, 2008). In this case, the fitness of an individual is expected to be best when developmental and adult environments match. For example, in humans, individuals that experienced nutritionally poor conditions at birth have been hypothesized to develop into a thrifty phenotype which has a greater propensity to store lipids and maintain high blood glucose (Barker et al., 1989; Gluckman et al., 2005; Leger et al., 1997). These phenotypic changes induced by early environment can be advantageous under nutritionally deprived adult environment, but may have detrimental consequences for fitness under affluent adult environment through causing diabetes and metabolic syndrome (Bateson et al., 2004; Gluckman & Hanson, 2004). Secondly, the “silver spoon” hypothesis holds that individuals developed or born in favorable environments have permanent fitness advantages over those grown under unfavorable environments (Grafen, 1988; Lindström, 1999). According to this hypothesis, poor environments experienced during development will constrain individuals from achieving high levels of fitness no matter whether their developmental and adult environments match or not. A recent update of this hypothesis, however, suggests that the extent of silver spoon effects can be either pronounced or suppressed depending on the quality of adult environments (Pigeon et al., 2019).

The quality of environmental conditions faced by individual animals in nature is characterized by a combination of multiple biotic and abiotic factors, which vary over time and space. Temperature and nutrition are among the most critical factors defining the quality of environmental conditions and animals are constantly challenged with combined stresses caused by inadequate temperature and nutrient imbalance under natural conditions (Cross et al., 2015; Rosenblatt & Schmitz, 2016). The profound impacts of thermal environment on individual fitness and life‐history traits are well established in ectothermic animals that rely on external heat source for regulating body temperature (Angilletta, 2009; Clarke, 2017). For example, a moderate increase in ambient temperature has been shown to result in accelerated juvenile growth, reduced body size at maturity, increased reproductive outcome, and shortened lifespan in insects (Kingsolver & Huey, 2008; Nunney & Cheung, 1997). Compared to those of temperature, the effects of nutrition on performance and fitness are more complex because nutrition has both qualitative and quantitative dimensions and includes multiple subcomponents that interact with each other in a complicated manner (Simpson & Raubenheimer, 2012). A large body of evidence accumulated over the past two decades has suggested that the balance between macronutrients (e.g., protein, carbohydrate, lipid) is the most important dietary determinant of performance and fitness in diverse organisms spanning slime molds to primates (reviewed in Simpson & Raubenheimer, 2012). For example, lifespan and reproduction have been shown to be dictated by dietary protein:carbohydrate (P:C) balance, with lifespan being maximized at a lower P:C ratio than that supports the maximum egg production rate in *Drosophila melanogaster* (Jang & Lee, 2018; Jensen et al., 2015; Lee, 2015; Lee et al., 2008) and in other insects (Fanson & Taylor, 2012; Harrison et al., 2014).

**Figure 1:**
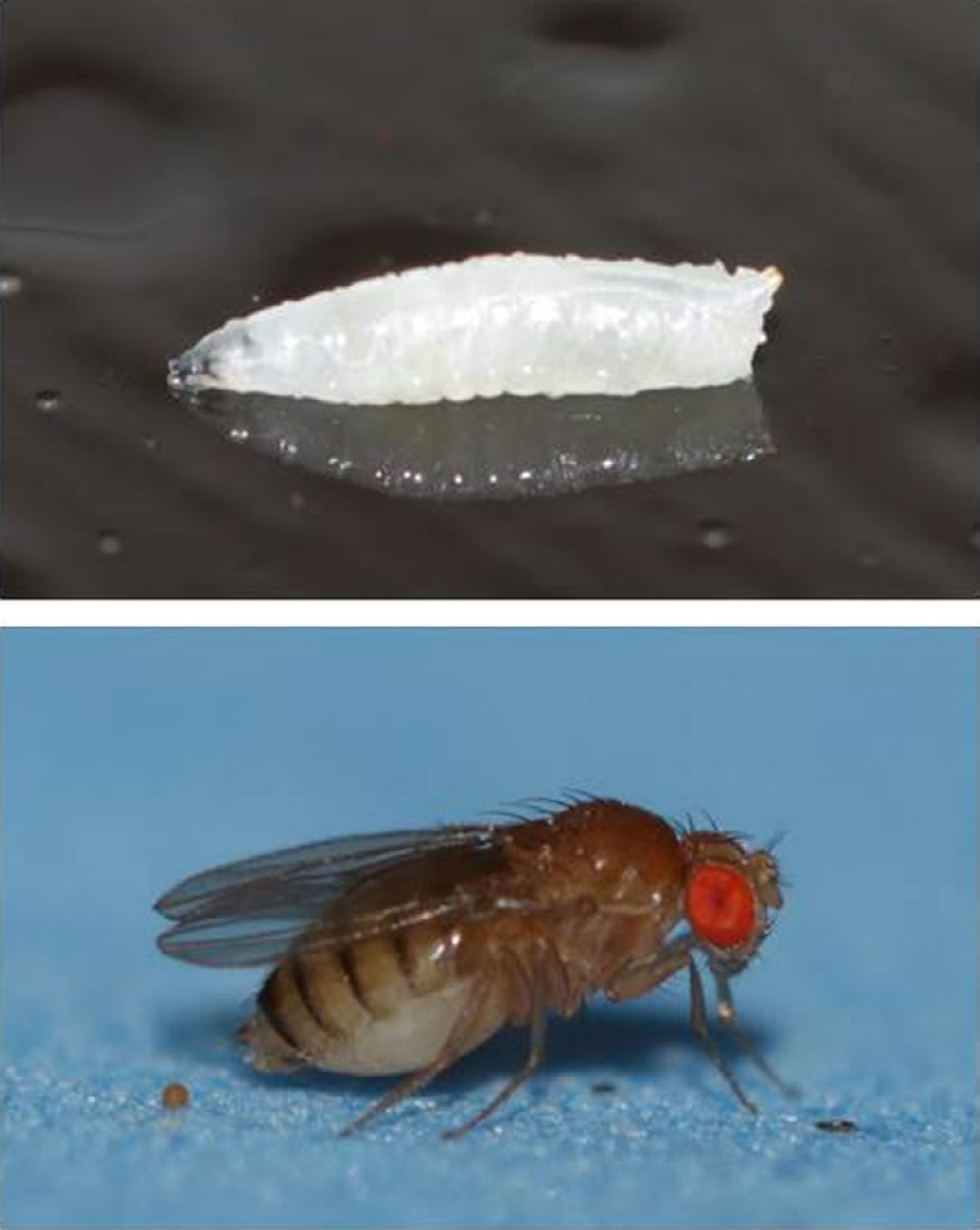
*Drosophila melanogaster* (above: larva; below: female adult). Photograph taken by K.P. Lee

The objective of this study was to explore the long‐term effects of environmental conditions experienced during the larval stage on adult survivorship and female reproductive success in the fruit fly, *D. melanogaster* Meigen (Diptera: Drosophilidae) (Figure 1). In this study, we experimentally manipulated the quality of environmental conditions by altering either the ambient temperature (Experiment 1) or dietary P:C balance (Experiment 2). We then compared whether and how these two environmental factors differed in the nature and magnitude of their long‐term effects on the key components of adult fitness. For each environmental factor, we employed a full factorial experimental design with varying levels of larval and adult environmental conditions. Apart from identifying the across‐stage effects of larval environments on adult life‐history traits, the application of this experimental design allowed us to test which of the two main hypotheses (environmental matching versus silver spoon hypothesis) would better explain the role of early growth conditions in shaping adult performance and fitness through examining the interactions between larval and adult environment conditions. If an adult individual performs better when exposed to an environment that is similar to that experienced during development than when exposed to a different one, we can take this result as evidence supporting the environmental matching hypothesis. On the other hand, the silver spoon hypothesis can be supported if adult performance is found to be constantly higher in individuals raised under favorable conditions (e.g., warm, nutritionally rich and balanced environments) compared to those raised under unfavorable ones (e.g., cold, nutritionally poor and unbalanced environments) regardless of the quality of adult environments.

## 2 MATERIAL AND METHODS

### 2.1 Experimental flies and husbandry

An outbred laboratory population of wild‐type Canton‐S strain *D. melanogaster* was used in this study (Figure 1). The laboratory stock population was derived from the Bloomington Stock Center (Indiana University, Indiana, USA) and had been maintained for several years on standard culturing medium (90.6 g dextrose, 68 g dry yeast, 42.8 g cornmeal, 6.5 g agar, 4.5 ml propionic acid and 1 g Nipagin in 1 L distilled water; henceforth, standard diet) at 25°C and 70% relative humidity (RH) under a 12 hr:12 hr light:dark cycle. To remove any undesired effects of parental and grandparental conditions on offspring phenotype, all experimental flies were raised at a consistent density of ca. 200–250 larvae in 150‐ml bottles for three consecutive generations prior to the start of this experiment. The protocol for achieving this consistent larval density across rearing bottles is described in Clancy and Kennington (2001).

### 2.2 Experimental design and setup

In this study, two separate experiments were performed to investigate the long‐term effects of larval temperature (Experiment 1) and nutrition (Experiment 2) on adult survivorship and fecundity. In Experiment 1, *D. melanogaster* larvae were raised at one of three larval temperatures (18, 23, or 28°C). For each larval temperature group, newly eclosed adults were randomly divided into three groups and each was allocated to one of three adult temperatures (18, 23, or 28°C). This resulted in a total of nine treatments representing the full combination of three larval and three adult temperatures. The experimental temperatures used in Experiment 1 are within the permissive range temperatures likely to be experienced by *D. melanogaster* in nature throughout their life cycle (Hoffmann, 2010). Throughout this experiment, larval and adult flies were maintained on the standard diet described above. Similarly, in Experiment 2, *D. melanogaster* larvae were raised on one of four chemically defined diets with differing protein:carbohydrate (P:C) ratio (1:8, 1:2, 2:1, or 8:1). Newly eclosed adult flies emerged from each larval diet were then divided into four groups, with each being assigned to one of four adult diets with differing P:C ratios (1:8, 1:2, 2:1, or 8:1). Thus, there were a total of sixteen treatments representing the full combination of four larval and four adult diets in Experiment 2. The dietary P:C ratios used in Experiment 2 were chosen based on the results of previous studies using the same chemically defined diets designed for *D. melanogaster* (Jang & Lee, 2018; Lee, 2015). Both larval and adult flies were kept at the constant temperature of 23°C throughout this experiment. All experiments were conducted in incubators (Vison Scientific Co., Ltd) set at 70% RH and a 12 hr:12 hr light:dark cycle. To control for any confounding effects of location within incubators, the position of fly vials in incubators was rotated more than three times a day.

### 2.3 Chemically defined diet

Chemically defined diets used in Experiment 2 were prepared following the protocol described in Lee (2015) and Jang and Lee (2018). These diets differed in P:C ratio (1:8, 1:2, 2:1, and 8:1), but contained the same concentration of protein plus carbohydrate (P + C = 120 g/L). Sodium caseinate (Sigma C8654) and sucrose (Sigma S9378) were used as the source of protein and carbohydrate, respectively. All diets used in this experiment comprised the same concentrations of dietary lipids (0.3 g/L cholesterol, 4 g/L lecithin), salt mixtures (0.71 g/L KH_2_PO_4_, 3.73 g/L K_2_HPO_4_, 0.62 g/L MgSO_4_, 1 g/L NaHCO_3_), nucleic acids (0.57 g/L uridine, 0.64 g/L inosine), vitamin mixtures (0.002 g/L thiamine, 0.01 g/L riboflavin, 0.012 g/L nicotinic acid, 0.0167 g/L calcium pantothenate, 0.0025 g/L pyridoxine, 0.0002 g/L biotin, 0.003 g/L folic acid), preservatives (1 g/L Nipagin, 0.3% propionic acid), and solidifying agent (20 g/L agar).

### 2.4 Protocol for assaying preadult life‐history traits

The measurement of preadult or larval life‐history traits required a large number of freshly laid eggs. These eggs were obtained by releasing approximately 1,000 newly eclosed male and female adults into a plastic egg laying cage (21 cm × 41 cm × 21 cm) where they were left to lay eggs for 4 hr on molasses‐agar plates (10% molassess fixed in 4% agar in 9‐cm diameter Petri dishes) seeded with live yeast paste (Jang & Lee, 2018). Eggs laid on this oviposition substrate were washed with 1 × phosphate‐buffered saline (PBS) and collected by filtering the resulting egg suspension through a nylon filter (~70 µm). Using a fine brush, we transferred these collected eggs to a strip of overhead projector (OHP) film (8 mm × 24 mm) which had a square grid (3 mm × 3 mm) printed on its surface. These transferred eggs were carefully placed inside each square grid and subsequently photographed using a high‐resolution DSLR camera (Canon EOS 600D; Canon Inc.) mounted with a macro lens (Canon EF 100 mm f/2.8 USM; Canon Inc.). Each film strip loaded with eggs was then transferred to a 20‐ml fly vial containing 7 ml of the experimental diet (standard diet in Experiment 1; chemically defined diet in Experiment 2). The exact number of eggs allocated to each vial was counted from the photographed images of the eggs. Through this method, an average of 50 eggs were distributed across replicate vials. Vials seeded with eggs were randomly divided into either three larval temperature treatments (Experiment 1; 40 vials for each) or four larval diet treatments (Experiment 2, 50 vials for each) and larvae hatched from these seeded eggs were allowed to develop until adult eclosion.

Preadult life‐history traits were measured from flies emerged from ten replicate vials per treatment. From each of these replicate vials, newly eclosed adults were collected at 3 hr intervals and the time of their emergence was recorded. Collected flies were frozen to death at −20°C, pooled across replicate vials, and sexed by inspecting the sexcomb. Only female flies were sorted out and randomly divided into 12 cohorts of five flies for each larval treatment. Cohorts were then dried at 65°C for three days before they were weighed to the nearest 1 µg using a BM‐22 analytical balance (A & D Co. Ltd.). The dry mass of individual flies was estimated as the dry mass of each cohort divided by five. Lipid in dried carcasses was extracted by soaking each cohort in 10 ml of diethyl ether for 24 hr. Lipid‐extracted samples were redried and reweighed. The difference in mass before and after the lipid extraction was taken as the measure of lipid content for each cohort. The lipid content of individual flies was estimated by dividing the lipid content of each cohort by five. Egg‐to‐adult viability was calculated as the percentage of eggs that successfully eclosed to adults in each vial. Preadult development time was determined as the time taken from eggs to adult eclosion for individual flies.

### 2.5 Protocol for assaying adult life‐history traits

Adult life‐history traits were recorded from flies emerged from the remaining vials not used for assaying preadult life‐history traits. To measure lifespan, newly eclosed adult flies that had been raised at one of three larval temperatures (Experiment 1) or on one of four larval diets (Experiment 2) were collected within 24 hr and housed in 150‐ml fly bottles containing 20 ml of standard diet (ca. 100–150 flies per bottle) where they were left to mate for 48 hr at 23°C. Mated males and females were separated under light CO_2_ anesthesia and only female flies were grouped into cohorts of ca. 30 individuals. Cohorts of female flies were transferred into 20‐ml fly vials containing 6 ml of the experimental diet (standard diet in Experiment 1; chemically defined diet in Experiment 2) and maintained at their respective experimental temperature (either 18, 23, or 28°C in Experiment 1; 23°C in Experiment 2). For each larval × adult treatment, there were four replicate vials, resulting in a total of ca. 100–120 flies. Deaths were scored daily and surviving flies were transferred to fresh vials every other day until no flies remained alive. To eliminate any adverse effects of dehydration on lifespan, the foam plugs of the fly vials were regularly moistened with distilled water during this lifespan assay.

Early‐life fecundity was determined by counting the number of eggs produced by triads of one female and two male flies housed in 20‐ml fly vials containing 4 ml of the experiment diet. Flies emerged from the same larval treatment were collected within 4 hr upon adult eclosion, sexed, and randomly grouped as triads. Fly triads were then randomly divided into adult treatments, with 15–20 replicate triads per larval × adult treatment. Fly triads were transferred into fresh vials every day and the number of eggs produced by each triad was counted daily for the first 10 days of adult life (days 0–10). Females that did not produce eggs or died during this experimental period were eliminated from the analysis.

### 2.6 Data analysis

All analyses were conducted using SAS v 9.12 statistical software (SAS Institute Inc.). The effect of larval environments (temperature or diet) on egg‐to‐adult viability, preadult development time, lean body mass, and lipid content at eclosion was analyzed using the general linear model (PROC GLM in SAS). The data for egg‐to‐adult viability were angular transformed prior to the analysis. When analyzing preadult development time, we included replicate vials as the random effect in the model. Cox proportional hazards regression (PROC PHREG) was used to analyze the separate and interactive effects of larval and adult environments (temperature or diet) on adult survivorship. The longitudinal pattern of daily egg production repeatedly measured from the same fly triads over the first 10 days of adult life (days 0–10) was analyzed using the generalized linear mixed model (PROC GLIMMIX), with the log link function and Poisson distribution. The model was fitted using larval and adult environments and age as the fixed effects. To account for the temporal pseudoreplication arising from taking repeated counts for the same fly triads, the identity of fly triads was designated as the random effect. Through this approach, we were able to separate within‐ and between individual effects on age‐specific pattern of egg production (van de Pol & Verhulst, 2006). Nonparametric LOWESS (locally weighted scatterplot smoothing using PROC LOWESS) with the smoothing parameter of 0.4 was applied to illustrate the pattern of daily egg production across days 0–10 for each treatment. The separate and interactive effects of larval and adult environments on the total number of eggs produced over days 0–5 and 5–10 were analyzed using the general linear model (PROC GLM). In these analyses, we calculated omega squared (ω^2^) as effect size to estimate the proportion of total variation in response variable (e.g., egg production) explained by a given factor (larval or adult environment).

## 3 RESULTS

### 3.1 Experiment 1: Long‐term effects of larval temperature

#### 3.1.1 Preadult life‐history traits

**Figure 2:**
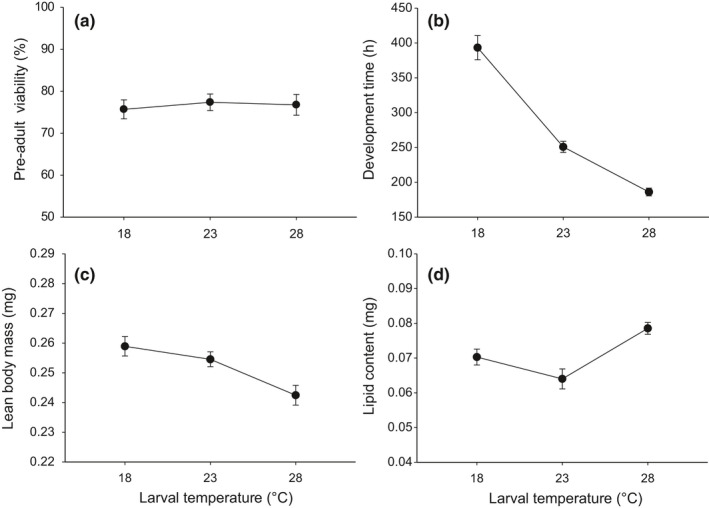
Effect of larval temperature on preadult life‐history traits: (a) egg‐to‐adult viability, (b) development time, (c) lean body mass, and (d) lipid content at eclosion in *D. melanogaster*. Body mass and lipid content were measured from females only. The values are mean ± 1 standard error of the mean (*SE*)

Egg‐to‐adult viability was maintained high (75.7%–77.2%) and was not significantly affected by the temperature experienced during the larval stage (larval temperature: *F*_2,26_ = 0.1, *p* = .904; Figure 2a). Preadult development time was significantly affected by larval temperature (*F*_2,1,158_ = 2,988.49, *p* < .001), with *D. melanogaster* exposed at 18 and 28°C exhibiting the longest (393.3 hr) and shortest development time (186.1 hr), respectively (Figure 2b). Larval temperature had a significant effect on the lean body mass of newly eclosed females (*F*_2,33_ = 7.65, *p* = .002). Female larvae raised at 18°C developed into adults with heavier lean body mass than those raised at higher temperatures (Figure 2c). The effect of larval temperature on lipid content recorded at adult eclosion was also significant (*F*_2,33_ = 9.22, *p* < .001), showing the highest lipid content at 28°C and the lowest at 23°C (Figure 2d).

#### 3.1.2 Adult survivorship

**Figure 3:**
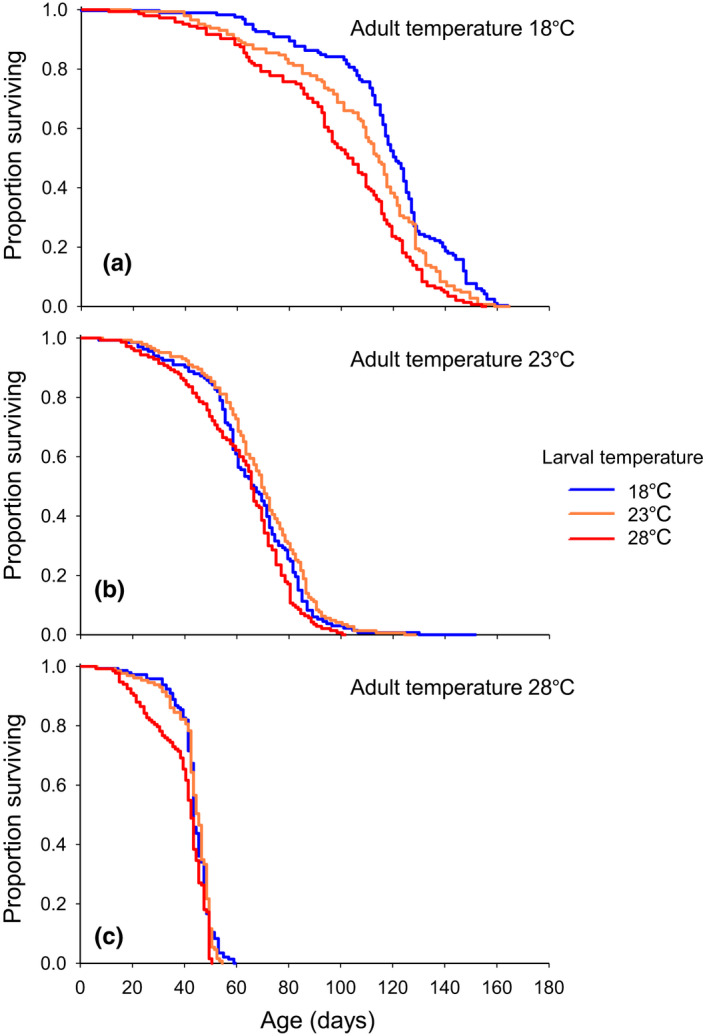
Effect of larval and adult temperature on adult survivorship for female *D. melanogaster*. Survivorship curves for *D. melanogaster* exposed to the adult temperature of 18, 23, and 28°C are presented in the panel (a), (b), and (c), respectively. In each panel, survivorship curves for *D. melanogaster* raised as larvae at 18, 23, and 28°C (larval temperature) are represented as blue, orange, and red lines, respectively

Adult survivorship was predominantly affected by the temperature experienced during the adult stage (adult temperature: *χ* ^2^ = 614.01, *df* = 2, *p* < .001). Survivorship was the highest when the temperature of adult environment was 18°C and reduced significantly as adult temperature increased (Figure 3). In accordance with this survivorship pattern, the median lifespan was the shortest and longest at the adult temperature of 28 and 18°C, respectively (Figure S1). The impact of larval temperature on adult survivorship was also significant but was much smaller compared to that of adult temperature (larval temperature: *χ* ^2^ = 8.27, *df* = 2, *p* < .016). Especially when adult temperature was 18°C, *D. melanogaster* raised at the larval temperature of 18°C exhibited higher survivorship (Figure 3) and, consequently, longer median lifespan (Figure S1) compared to those raised at higher larval temperatures. There was no significant interaction between larval and adult temperature for adult survivorship (*χ* ^2^ = 7.24, *df* = 4, *p* = .124).

#### 3.1.3 Early‐life fecundity

**Figure 4:**
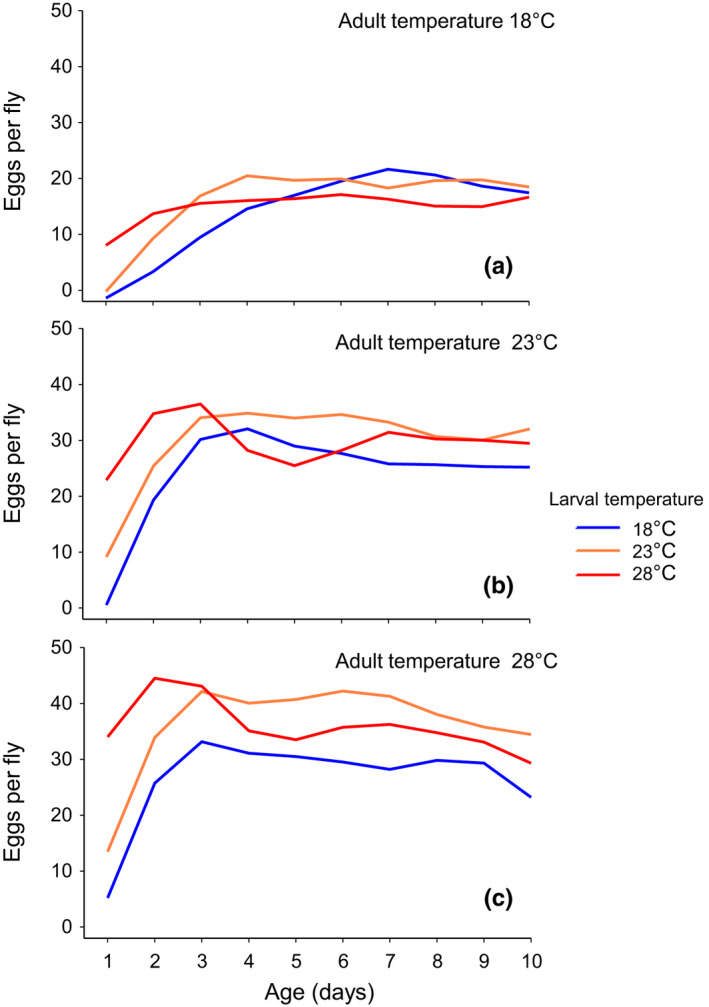
Effect of larval and adult temperature on daily egg production (the number of eggs produced per day) recorded across days 0–10 posteclosion for female *D. melanogaster*. The longitudinal patterns of daily egg production for *D. melanogaster* exposed to the adult temperature of 18, 23, and 28°C are presented in the panel (a), (b), and (c), respectively. In each panel, the patterns of daily egg production for *D. melanogaster* raised as larvae at 18, 23, and 28°C (larval temperature) are represented as blue, orange, and red lines, respectively

The number of eggs produced per day (daily egg production) over the first 10 days posteclosion (days 0–10, henceforth) was significantly affected by the temperature of both adult and larval environments (Table 1). Regardless of larval temperature, *D. melanogaster* exposed to higher adult temperatures produced more eggs than those exposed to lower adult temperatures (Figure 4). Daily egg production was lower in *D. melanogaster* raised as larvae at 18°C compared to those raised at higher larval temperatures (Figure 4). The effect of age was also significant for daily egg production (Table 1), suggesting an age‐dependent change in daily egg production. However, as indicated by significant two‐way and three‐way interactions between the age and the temperature of both larval and adult environments (Table 1), the way in which daily egg production changed with age differed depending on the temperature of larval and adult environments. When the temperature encountered during the adult stage was 23 or 28°C, for example, daily egg production increased rapidly until day 3 posteclosion (30–45 eggs per day), after which it stabilized (Figure 4b,c). In contrast, when adult temperature was 18°C, daily egg production remained low (20 eggs per day) and increased slowly with age (Figure 4a). At all three adult temperatures, *D. melanogaster* raised at 28°C tended to lay more eggs than those raised at 23°C until day 3 posteclosion, but the trend was reversed thereafter.

**Table 1:** Summary of the generalized linear mixed model examining the effect of larval and adult temperature and fly age on the repeated measures of daily egg production by female *D. melanogaster* across days 0–10 posteclosion

Source	Num *df*	Den *df*	*F*	*p*
Larval temperature (LT)	2	1,404	60.81	<.001
Adult temperature (AT)	2	1,404	227.51	<.001
LT × AT	4	1,404	1.55	.184
Age	1	1,404	414.52	<.001
Age × LT	2	1,404	129.82	<.001
Age × AT	2	1,404	111.97	<.001
Age × LT×AT	4	1,404	7.72	<.001

**Figure 5:**
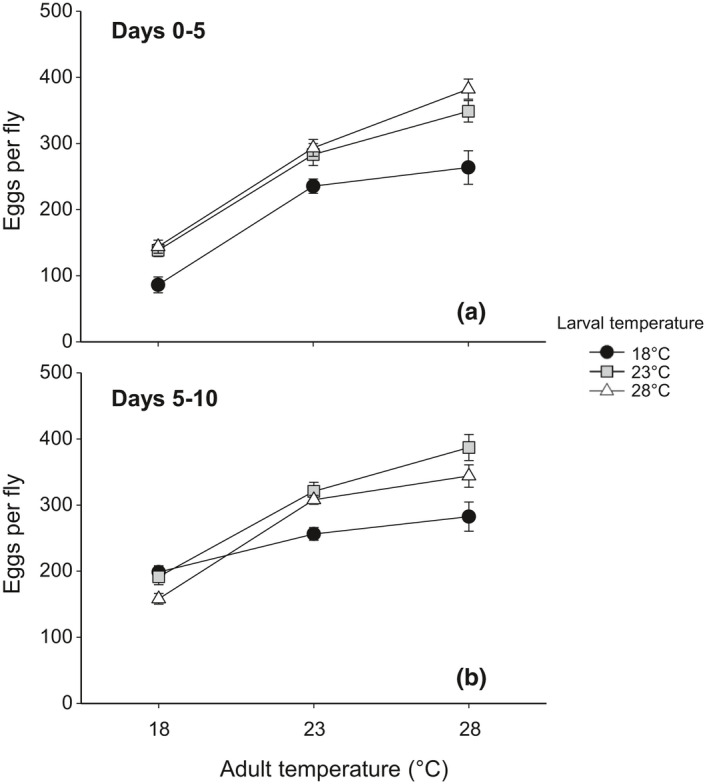
Effect of larval and adult temperature on the total number of eggs produced by female *D. melanogaster* over (a) days 0–5 and (b) days 5–10 posteclosion. The values are mean ± 1 standard error of the mean (*SE*)

To further understand whether the relative contribution of larval and adult temperature to egg production would change over the course of the early adult stage, we conducted separate analyses on the effects of larval and adult temperature on the cumulative number of eggs produced by the same females over the first (days 0–5; Figure 5a) and the second five‐day period (days 5–10; Figure 5b) since adult eclosion. The number of eggs produced over days 0–5 was significantly affected by the temperature of both larval and adult environments (Table 2). The number of eggs produced over days 0–5 increased progressively as the temperature exposed during the adult stage increased from 18 to 28°C (Figure 5a). The adult temperature effect accounted for ca. 51.2% of the total variation in egg production over days 0–5 (Table 2). Compared to this strong adult temperature effect, the effect of larval temperature on egg production over days 0–5 was much smaller, explaining only 6.2% of its total variation (Table 2). *Drosophila melanogaster* raised at the lowest larval temperature of 18°C produced consistently fewer eggs over days 0–5 than those raised at 23 and 28°C regardless of adult temperature (Figure 5a). Post hoc tests revealed that egg production over days 0–5 was not significantly different between flies raised at 23 and 28°C (Tukey HSD test: *p* > .05). The magnitude of this negative effect of low larval temperature on adult fecundity tended to decrease with decreasing adult temperature (Figure 5a), but the interaction between larval and adult temperature was not significant (explaining < 0.4% of the total variation; Table 2).

**Table 2:** Summary of the general linear model examining the effect of larval and adult temperature on the total number of eggs produced by female *D. melanogaster* over days 0–5 and days 5–10 posteclosion

Period	Source	*df*	*F*	*p*	*ω* ^2^
Days 0–5	Larval temperature (LT)	2	14.37	<.001	0.0620
	Adult temperature (AT)	2	111.53	<.001	0.5124
	LT × AT	4	1.41	.232	0.0038
	Error	148			
Days 5–10	LT	2	8.43	<.001	0.0419
	AT	2	65.3	<.001	0.3626
	LT × AT	4	2.83	.027	0.0206
	Error	148			

The overall pattern of the number of eggs produced over days 5–10 was qualitatively similar to that described over days 0–5 (Figure 5b), with the only exception being that the interaction between larval and adult temperature was statistically significant (Table 2). Whereas the adult temperature effect explained ca. 36.3% of the total variation in the total number of eggs produced over days 5–10, the larval temperature effect explained only ca. 4.2% (Table 2). These results suggest that the contribution of larval temperature to egg production was consistently lower than that of adult temperature throughout the early adult life in *D. melanogaster*.

### 3.2 Experiment 2: Long‐term effects of larval nutrition

#### 3.2.1 Preadult life‐history traits

**Figure 6:**
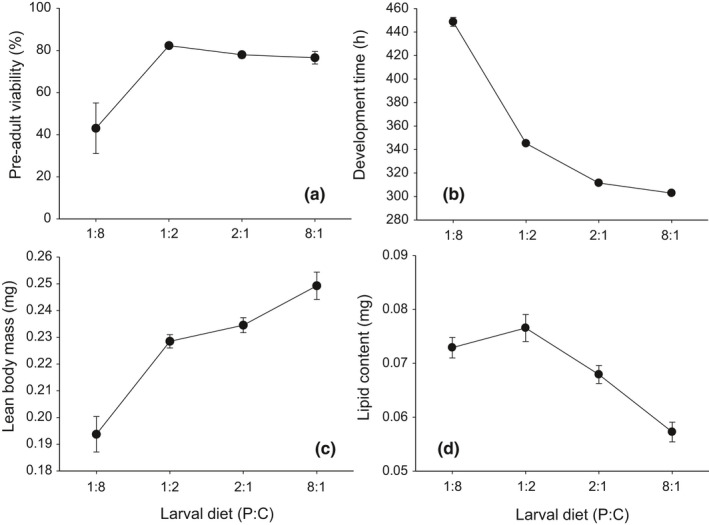
Effect of larval diet on preadult life‐history traits: (a) egg‐to‐adult viability, (b) development time, (c) lean body mass, and (d) lipid content at eclosion in *D. melanogaster*. Body mass and lipid content were measured from females only. The values are mean ± 1 standard error of the mean (*SE*)

The P:C ratio of the diet consumed by *D. melanogaster* during their larval stage (larval diet, henceforth) had a significant effect on egg‐to‐adult viability (*F*_3,34_ = 7.36, *p* < .001). *Drosophila melanogaster* raised on a diet with the lowest P:C ratio of 1:8 exhibited a substantially reduced egg‐to‐adult viability (43.0%) compared to those raised on other diets (76.5%–82.3%) (Figure 6a). Post hoc test revealed that egg‐to‐adult viability was not statistically different among flies raised on diets with three high P:C ratios (1:2, 2:1, and 8:1) (Tukey HSD test: *p* > .05). The effect of larval diet on preadult development time was also significant (*F*_3,1,229_ = 2,568.25, *p* < .001). Development time was the shortest in *D. melanogaster* raised on the most protein‐biased diet (P:C 8:1) and delayed progressively as the dietary P:C ratio of larval diet decreased (Figure 6b). The lean body mass of females at adult eclosion was significantly affected by larval diet (*F*_3,44_ = 26.2, *p* < .001), exhibiting a gradual increase as a function of increasing dietary P:C ratio (Figure 6c). The effect of larval diet was also significant for lipid content (*F*_3,44_ = 17.74, *p* < .001). *Drosophila melanogaster* raised on two diets with low P:C ratios (1:8 and 1:2) accumulated substantially more lipids in their body than those raised on a diet with the highest P:C ratio of 8:1 (Figure 6d).

#### 3.2.2 Adult survivorship

**Figure 7:**
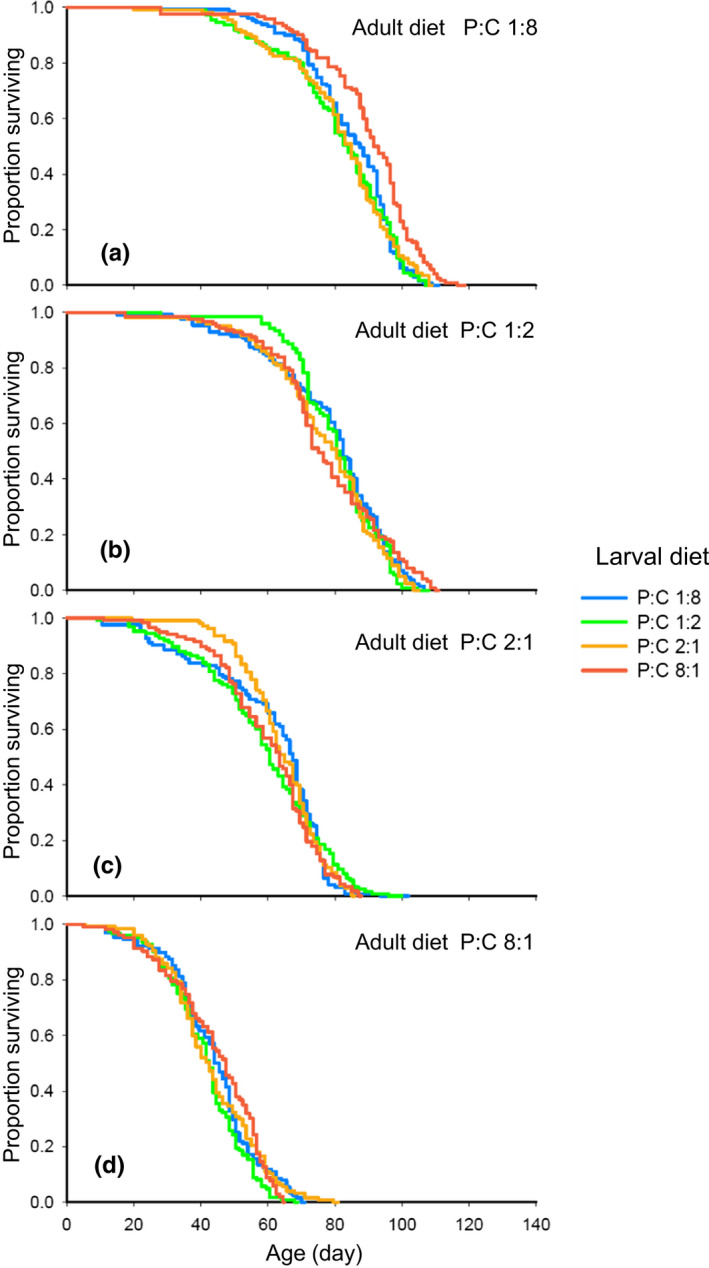
Effect of larval and adult diet on adult survivorship for female *D. melanogaster*. Survivorship curves for *D. melanogaster* fed on the adult diet with P:C ratio 1:8, 1:2, 2:1 and 8:1 are presented in the panel (a), (b), (c), and (d), respectively. In each panel, survivorship curves for *D. melanogaster* raised on the larval diet with P:C 1:8, 1:2, 2:1 and 8:1 are represented as blue, green, orange, and red lines, respectively

The survivorship of adult female *D. melanogaster* was profoundly affected by the P:C ratio of the diet consumed during their adult stage (adult diet, henceforth) (*χ* ^2^ = 576.39, *df* = 3, *p* < .001), with the ingestion of adult diets with high P:C ratio leading to reduced survivorship (Figure 7). Accordingly, the median lifespan was the shortest and longest at the adult P:C ratio of 8:1 and 1:8, respectively (Figure S2). Despite being much smaller than that of adult diet, the effect of larval diet on survivorship was also significant (*χ* ^2^ = 22.86, *df* = 3, *p* < .001). However, a significant interaction between larval and adult diet (*χ* ^2^ = 26.20, *df* = 9, *p* = .002) indicated that the effect of larval diet on adult survivorship differed depending on adult diet. For example, *D. melanogaster* raised on a larval diet with the P:C ratio of 8:1 exhibited higher survivorship and thus longer median lifespan compared to those raised at the other ratios when the P:C ratio of adult diet was 1:8 (*χ* ^2^ = 25.16, *df* = 3, *p* < .001; Figure 7a). We did not detect any significantly different effects across larval diets on adult survivorship when the P:C ratio of adult diet was 1:2, 2:1, and 8:1 (P:C 1:2: *χ* ^2^ = 1.97, *df* = 3, *p* = .578; P:C 2:1: *χ* ^2^ = 0.97, *df* = 3, *p* = .804; P:C 8:1: *χ* ^2^ = 7.11, *df* = 3, *p* = .068; Figure 7b–d).

#### 3.2.3 Early‐life fecundity

**Figure 8:**
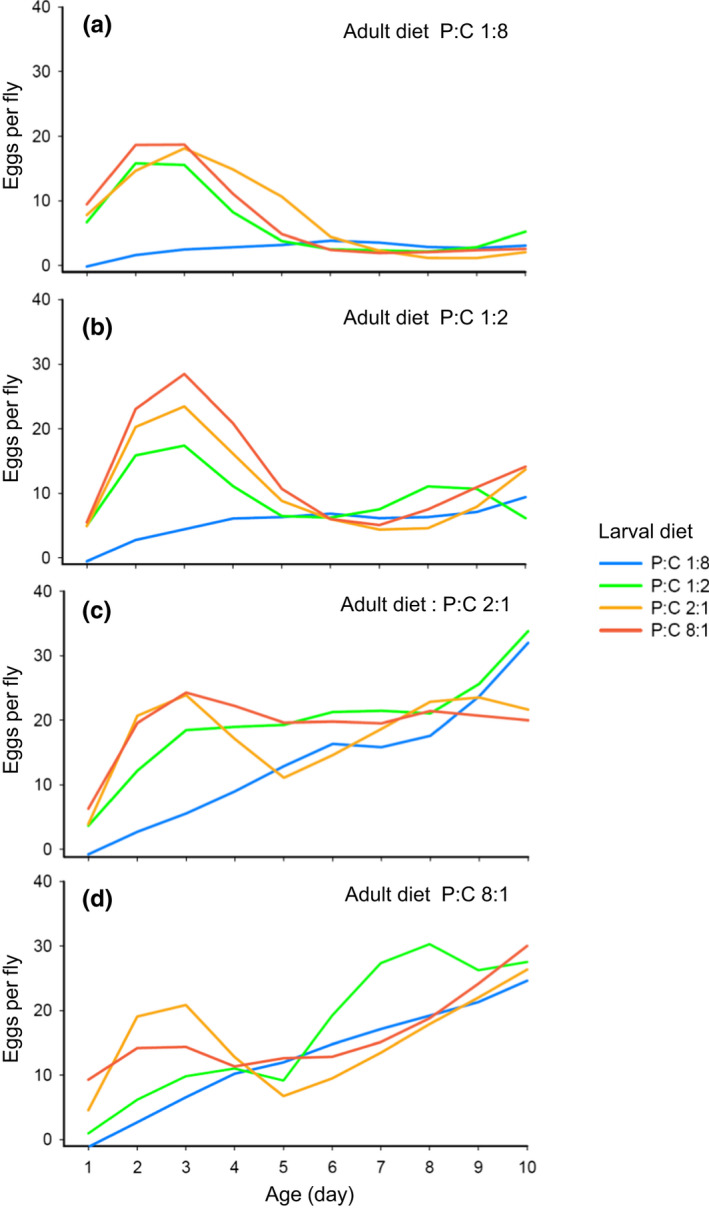
Effect of larval and adult diet on daily egg production (the number of eggs produced per day) recorded across days 0–10 posteclosion for female *D. melanogaster*. The longitudinal patterns of daily egg production for *D. melanogaster* fed on the adult diet with P:C 1:8, 1:2, 2:1 and 8:1 are presented in the panel (a), (b), (c), and (d), respectively. In each panel, the patterns of daily egg production for *D. melanogaster* raised on the larval diet with P:C 1:8, 1:2, 2:1 and 8:1 are represented as blue, green, orange, and red lines, respectively

The number of eggs produced per day (daily egg production) changed significantly with age and this age‐dependent change in daily egg production varied according to the P:C ratio of both larval and adult diets, as indicated by significant effects of age and two‐way and three‐way interactions between age and the two diets (Table 3). Regardless of adult diet, the daily egg production of *D. melanogaster* raised on larval diets with three high P:C ratios (1:2, 2:1, or 8:1) increased rapidly over days 0–3 and thereafter fell. However, no such early peaking pattern was observed for those raised on a larval diet with the lowest P:C ratio of 1:8 (Figure 8). The daily egg production of *D. melanogaster* raised on the most protein‐deficient diet (P:C 1:8) was strongly suppressed throughout the first 10 days of adult stage when the P:C ratio of adult diet was 1:8, but increased gradually with age when the P:C ratio of adult diet was 1:2, 2;1, or 8:1 (Figure 8). After day 5 posteclosion, the daily egg production of *D. melanogaster* raised on all four larval diets converged to a level that was almost indistinguishable to each other.

**Table 3:** Summary of the generalized linear mixed model examining the effect of larval and adult diet and fly age on the repeated measures of daily egg production by female *D. melanogaster* across days 0–10 posteclosion

Source	Num *df*	Den *df*	*F*	*p*
Larval diet (LD)	3	2,603	81.04	<.001
Adult diet (AD)	3	2,603	29.09	<.001
LD × AD	9	2,603	2.38	.011
Age	1	2,603	70.66	<.001
Age × LD	3	2,603	56.63	<.001
Age × AD	3	2,603	171.43	<.001
Age × LD ×AD	9	2,603	3.17	<.001

**Figure 9:**
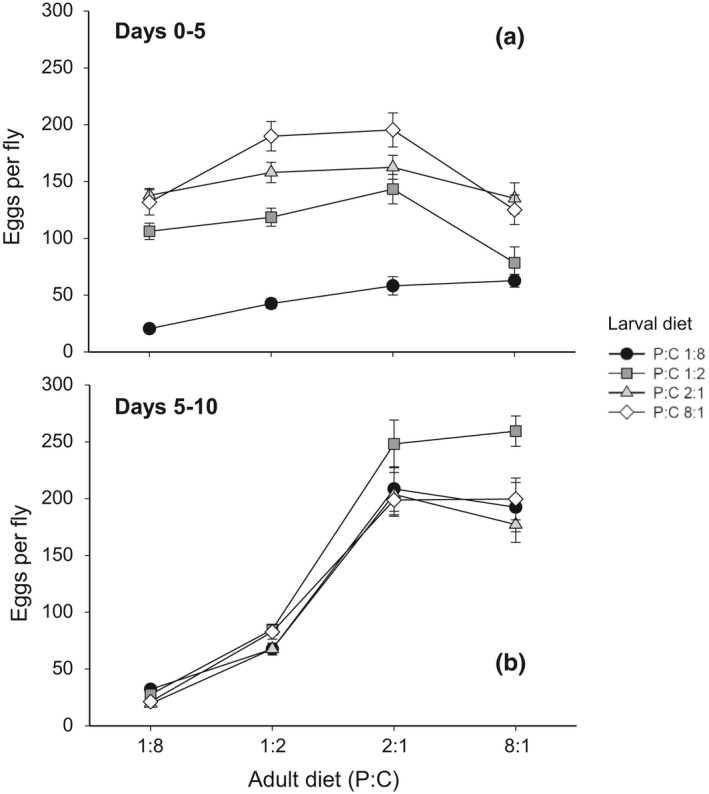
Effect of larval and adult diet on the total number of eggs produced by female *D. melanogaster* over (a) days 0–5 and (b) days 5–10 posteclosion. The values are mean ± 1 standard error of the mean (*SE*)

For the reason similar to Experiment 1, the effects of larval and adult diets on the number of eggs produced over days 0–5 and days 5–10 were analyzed separately (Figure 9). The number of eggs produced over these two five‐day periods was significantly influenced by both larval and adult diets (Table 4), but the way in which these diets influenced egg production differed qualitatively between these two periods. The pattern of egg production over days 0–5 was more strongly affected by larval diet than by adult diet. The effect of larval diet explained ca. 45.8% of the total variation in egg production over days 0–5 while that of adult diet explained only 6.6% (Table 4). Regardless of the P:C ratio of adult diet, *D. melanogaster* raised on larval diets with lower P:C ratios produced fewer eggs over days 0–5 than those raised on larval diets with higher P:C ratios (Figure 9a).

**Table 4:** Summary of the general linear model examining the effect of larval and adult diet on the total number of eggs produced by female *D. melanogaster* over days 0–5 and days 5–10 posteclosion

Period	Source	*df*	*F*	*p*	*ω* ^2^
Days 0–5	Larval diet (LD)	3	103.25	<.001	0.4581
	Adult diet (AD)	3	15.73	<.001	0.0660
	LD × AD	9	3.22	.001	0.0299
	Error	275			
Days 5–10	LD	3	7.22	<.001	0.0186
	AD	3	226.4	<.001	0.6743
	LD × AD	9	1.73	.083	0.0065
	Error	275			

In a manner completely opposite to that observed for egg production over days 0–5, the P:C ratio of adult diet had a strong effect on the number of eggs produced over days 5–10, explaining ca. 67.4% of the total variation in egg production over days 5–10 (Table 4). The number of eggs laid over days 5–10 was the lowest in *D. melanogaster* fed on an adult diet with the lowest P:C ratio of 1:8 and gradually increased as the P:C ratio of adult diet increased from 1:8 to 2:1 (Figure 9b). Strong carry‐over effect of larval diet observed over days 0–5 diminished rapidly to such an extent that it explained only 1.9% of the total variation in egg production over days 5–10 (Table 4). These results suggest that there was a major shift in the diet contributing mainly to egg production from larval to adult diet after day 5 posteclosion.

## 4 DISCUSSION

We found that *D. melanogaster* raised on a diet with the lowest P:C ratio of 1:8 suffered high preadult mortality, delayed development, and reduced body mass at eclosion. This result is largely consistent with those of previous *D. melanogaster* studies (Gray et al., 2018; Jang & Lee, 2018; Rodrigues et al., 2015) and is likely to be driven by the limited intake of dietary protein, which provides the raw materials for somatic growth and also stimulates the release of insulin‐like growth factors promoting cellular growth and proliferation in *D. melanogaster* (Géminard et al., 2009; Mirth et al., 2019). Previous studies have repeatedly demonstrated that ectothermic animals, including insects, grow more slowly but attain a larger body size at maturity when raised under cooler environments, a phenomenon widely known as the temperature‐size rule for ectotherms (Atkinson, 1994; Kingsolver & Huey, 2008). Our results confirmed that this rule was followed by *D. melanogaster*, as has been previously described (Nunney & Cheung, 1997; Partridge et al., 1994).

Our results showed that exposure to high temperatures during the adult stage resulted in reduced adult survivorship and shortened lifespan, which is in accordance with the well established pattern of temperature‐dependence of lifespan previously described in *D. melanogaster* (Bochdanovits & de Jong, 2003; Miquel et al., 1976; Nunney & Cheung, 1997; Zwaan et al., 1992). This negative effect of high temperature on adult survivorship may be attributed to accelerated kinetics of biological reactions, decreased metabolic efficiencies, increased molecular damages caused by elevated production of reactive oxygen species, and/or altered 4E‐BP growth pathway (Carvalho et al., 2017; Conti, 2008; Keil et al., 2015; Sestini et al., 1991). Despite being not as strong as the direct effect of adult temperature, we also found that the temperature experienced during the larval stage had a lasting effect on adult survivorship, showing that *D. melanogaster* raised under cool larval environments lived longer as adults than those raised under warm environments. Extended longevity for adult individuals raised under cool larval environments has been documented from *D. melanogaster* and other holometabolous insects (Lints & Lints, 1971; Cohet, 1975; Christiansen‐Jucht et al., 2014; but see Economos & Lints, 1986; Zwaan et al., 1992) and could be attributed to their larger body size, given the positive association reported between body size and longevity in *D. melanogaster* (Partridge & Farquhar, 1981; McCabe & Partridge, 1997; but see Zwaan et al., 1992). An alternative but not mutually exclusive possibility is that extended lifespan observed from *D. melanogaster* developed in cold environments is the outcome of their physiological and metabolic acclimations to cold environments encountered during development. An increase in sugars, polyols, and free amino acids has been reported in *D. melanogaster* raised at cold temperatures and is known to confer insects with improved cold tolerance (Colinet & Hoffmann, 2012; Koštál et al., 2011). Since longevity is positively correlated with physiological tolerance to cold stress in *D. melanogaster* (Luckinbill, 1998), it is possible that any physiological changes that improved cold tolerance could have also led *D. melanogaster* to live longer.

Our results showed that the consumption of a diet with high P:C ratio during the adult stage reduced adult survivorship in *D. melanogaster*, as has been previously described (Davies et al., 2018; Jang & Lee, 2018; Jensen et al., 2015; Lee, 2015; Lee et al., 2008). This life‐shortening effect of high P:C intake is likely to be mediated through elevated nitrogenous waste products, increased mitochondrial reactive oxygen species, and/or increased activation of mTOR signaling pathways (reviewed by Le Couteur et al., 2016; Mirzaei et al., 2014; Simpson et al., 2017). While there are ample studies describing the role of adult macronutrient consumption in shaping adult survivorship, empirical evidence is relatively scarce for the long‐term effects of larval macronutrient consumption on adult survivorship in *D. melanogaster* and other insects (Davies et al., 2018; but see Runagall‐McNaull et al., 2015; Duxbury & Chapman, 2020). Here, we report that the P:C ratio of the diet consumed during the larval stage can have a small but lasting effect on adult survivorship. For example, *D. melanogaster* raised on a larval diet with the highest P:C ratio of 8:1 exhibited improved adult survivorship compared to those raised on the other diets when the P:C ratio of adult diet was 1:8. A possible explanation for this result is that high protein intake during development might have produced an adult phenotype with enhanced somatic repair and maintenance mechanisms (Runagall‐McNaull et al., 2015) or with altered aging‐related nutrient sensing pathways (Pasco & Léopold, 2012; Pooraiiouby et al., 2018). However, it still remains to be explained why this life‐extending effect of high P:C ratio occurred only when the P:C ratio of adult diet was 1:8. It is possible that the extreme carbohydrate deficiency experienced by *D. melanogaster* raised at P:C 8:1 has induced a “thrifty phenotype” which is programmed to conserve and store ingested carbohydrates more efficiently in preparation for carbohydrate‐depriving condition anticipated later in life (Bateson et al., 2004; Gluckman et al., 2005; Hales & Barker, 1992). This diet‐induced propensity to conserve energy may lead *D. melanogaster* to accumulate excessively high levels of energetic substrates (in form of trehalose, glycogen, and lipid) in their body if they are unexpectedly exposed to a high‐carbohydrate adult environment. While the mismatch between poor developmental nutrition and affluent adult nutrition has been generally considered to have detrimental effects on adult health and fitness in humans (Barker et al., 1989; Gluckman & Hanson, 2004; Leger et al., 1997), it might have played a role in extending lifespan in *D. melanogaster* instead, given that body lipid content and lifespan are positively correlated in this species (Hansen et al., 2013).

It is well established that the number of eggs produced by female *D. melanogaster* during early adulthood increases as the temperature of adult environment rises moderately (Huey et al., 1995; Klepsatel et al., 2013) and as the P:C ratio of the diet consumed during the adult stage increases (Jang & Lee, 2018; Jensen et al., 2015; Lee, 2015; Lee et al., 2008). This general pattern of temperature‐ and nutrient‐dependence of female fecundity was corroborated in this study. In addition to these direct effects, we found that the temperature and nutrition of larval environments had significant impacts on early female fecundity in *D. melanogaster*. Since body mass is positively associated with fecundity in insects (Honěk, 1993), we initially predicted that larger flies emerged from cooler larval environments would produce more eggs. Rather unexpectedly, however, our data showed that female *D. melanogaster* raised at 18°C produced constantly fewer eggs over the early adult stage than those raised at warmer temperatures (23 and 28°C), suggesting that the temperature‐induced increase in body size does not necessarily lead to an increase in fecundity in *D. melanogaster* (Huey et al., 1995; Klepsatel et al., 2019; Nunney & Cheung, 1997). Then, what could be the mechanism underlying the negative effect of cool developmental temperature on adult fecundity? In *D. melanogaster*, ovariole number is a key morphological feature that is positively correlated with daily egg production (Boulétreau‐Merle et al., 1982; Klepsatel et al., 2013). Numerous studies have repeatedly demonstrated that the number of ovarioles is significantly reduced in *D. melanogaster* reared in cool environments, suggesting that the temperature experienced during larval development is a major environmental determinant of ovariole number in *D. melanogaster* (Cohet & David, 1978; Delpuech et al., 1995; Hodin & Riddiford, 2000; Klepsatel et al., 2013, 2019; Sarikaya et al., 2012). Although it needs to be validated in the future, these previous results lead us to predict that *D. melanogaster* raised at 18°C would have fewer ovarioles than those raised at higher temperatures. It is also reported that, once it is fixed during the late stage of larval development, ovariole number does not change in response to variations in environmental conditions experienced after metamorphosis (Hodin & Riddiford, 2000), perhaps explaining why the negative effect of cool rearing temperature on adult egg production lasted throughout the early adult stage in this study. Another likely possibility is that reduced egg production for cool‐reared *D. melanogaster* may be the outcome of a trade‐off with higher tolerance to cold environments. Multiple studies have reported that acclimation to low temperature results in increased tolerance to cold stress at the expense of reduced reproductive performance in *D. melanogaster* and other insects (Bubliy et al., 2002; Coulson & Bale, 1992; Hoffmann, 1995; Jensen et al., 2017; Overgaard et al., 2007).

There are numerous studies documenting reduced or suppressed egg production in *D. melanogaster* fed on diets with restricted protein content (Jang & Lee, 2018; Jensen et al., 2015; Lee et al., 2008), representing the prime importance of dietary protein as raw materials for producing eggs in this species and other insects (Mirth et al., 2019). Our results were consistent with those of previous studies, showing that egg production was improved in *D. melanogaster* that were fed on diets with high P:C ratio as both larvae and adults. Over the first five days posteclosion (days 0–5), *D. melanogaster* reared on a diet with the lowest P:C ratio (1:8) produced significantly fewer eggs than those reared on diets with higher P:C ratios, indicating that egg production during this early adult stage was constrained by poor protein nutrition carried over from the larval stage (Tu & Tatar, 2003). As income breeders, *D. melanogaster* mainly use nutrients acquired from their adult diets to produce eggs; however, even for them, the contribution of larval‐derived nutrition to egg production has been shown to be substantial during the first few days after adult eclosion (Aguila et al., 2013). Accordingly, it is reasonable to expect that the amount of larval‐derived proteins that can be later used for producing eggs will be low in *D. melanogaster* raised on a larval diet with limited protein content (Boggs, 2009). This strong effect of larval diet on egg production however diminished after day 5 posteclosion and was subsequently replaced by that of adult diet. Collectively, these results suggest that the main proteinaceous resources allocated to egg production switched from those derived from larval diets to those from adult diets after day 5 posteclosion in *D. melanogaster*. Similar temporal transition in nutrient allocations to female fecundity has been reported from other income breeding holometabolous insects, such as *Dinarmus basalis* (Rivero et al., 2001).

Nunney and Cheung (1997) reported that early female fecundity was higher in *D. melanogaster* when developmental and adult temperature matched than when they did not, suggesting the thermally induced adaptive phenotypic plasticity in this species. Unlike their results, we did not find any evidence to support that the matching between development and adult environments leads to improved adult performance in *D. melanogaster*. This was not unexpected since the empirical evidence for this environmental matching hypothesis has been rather sparse (Monaghan, 2008; Pigeon et al., 2019). On the contrary, especially when the reproductive outcome is concerned, we observed that *D. melanogaster* raised as larvae in thermally and nutritionally favorable environments outperformed those raised in unfavorable environments, no matter whether early and adult environments matched or not. These results suggest that any advantages obtained during development will bring lifelong fitness benefits to individuals, thereby providing support for the silver spoon hypothesis (Grafen, 1988; Lindström, 1999; Monaghan, 2008).

The presence of a silver spoon effect on reproduction has been previously demonstrated in *D. melanogaster* and other insects (Bauerfeind & Fischer, 2005; Boggs & Freeman, 2005; Dmitriew & Rowe, 2011; Tu & Tatar, 2003), but the most important and novel finding of this study is that the nature, strength, and persistence of this silver spoon effect differ substantially depending on the type of environmental factor characterizing the quality of early or developmental environments. For example, the effect of larval temperature on adult egg production was weak, permanent, and irreversible whereas that of larval nutrition was strong, transient, and reversible. These contrasting differences between these two aspects of early environments lead us to hypothesize that the thermal history of an individual may impose a more robust and longer lasting constraint on reproduction and perhaps fitness than the nutritional history in *D. melanogaster*. Why these differences emerge remains enigmatic, but may reflect fundamental differences in the way in which physiology, metabolism, and life‐history traits are controlled by these two factors.

In the present study, we have investigated the impacts exerted by thermal and nutritional environments experienced during development on adult performance separately. In reality, however, temperature and nutrition do not operate independently because they are tightly connected to each other via metabolism and energy requirement (Clissold & Simpson, 2015; Cross et al., 2015). Accordingly, there has been a growing need to integrate two these factors into a common framework and we have recently begun to gain a deeper knowledge of how the interactions between temperature and nutrition can shape organismal fitness in ectotherms through affecting their physiology, behavior, and life‐history traits (Chakraborty et al., 2020; Kim et al., 2020; Kurtz et al., 2019; Lee et al., 2015). A logical next step for future research would therefore be to examine how the thermal and nutritional histories experienced during development interact to affect adult lifespan and reproductive outcome.

In summary, we show that the environments experienced during early life have significant repercussions on the reproductive outcome of *D. melanogaster*. This highlights the importance of the thermal and nutritional histories of an individual in shaping its organismal phenotype and fitness. Our data also experimentally demonstrate that development under thermally and nutritionally favorable conditions confers lifelong fitness advantages in *D. melanogaster*, providing support for the silver spoon hypothesis. More importantly, we report a major difference in the nature, strength, and persistence of these silver spoon effects between thermal and nutritional environments experienced during development. How such difference between thermal and nutritional histories can translate into different ecological consequences should be addressed in future research. Since ectotherms are faced with growing challenges posed by anthropogenic changes in thermal and nutritional environments (Rosenblatt & Schmitz, 2016; Smith & Myers, 2018; Snell‐Rood et al., 2015), we envisage that the results obtained in this work will have valuable implications for improving our predictive understanding of the responses of ectotherms to environmental changes.

## CONFLICT OF INTEREST

The authors declare that they have no conflict of interest.

## AUTHOR CONTRIBUTIONS

**Kyeong Woon Min:** Conceptualization (lead); Formal analysis (lead); Investigation (lead); Methodology (lead); Validation (lead); Visualization (lead); Writing‐original draft (lead). **Taehwan Jang:** Conceptualization (supporting); Investigation (supporting); Methodology (supporting); Resources (equal); Software (equal); Validation (equal); Visualization (supporting); Writing‐original draft (supporting); Writing‐review & editing (supporting). **K.P. Lee:** Conceptualization (lead); Data curation (lead); Formal analysis (lead); Funding acquisition (lead); Investigation (lead); Methodology (lead); Project administration (lead); Resources (lead); Software (lead); Supervision (lead); Validation (lead); Visualization (lead); Writing‐original draft (lead); Writing‐review & editing (lead).

## Supporting information

Fig S1‐S2

## Data Availability

The data are available in the Dryad Data Repository (https://doi.org/10.5061/dryad.jsxksn07p).
