# Supplementary material for: Thermal and nutritional environments during development exert different effects on adult reproductive success in Drosophila melanogaster
Source: Ecol Evol. 2020 Nov 24;11(1):443–57. doi: 10.1002/ece3.7064 (PMC7790642; doi:10.1002/ece3.7064)
Supplement: Supplementary file 1 — Fig S1‐S2 [file ECE3-11-443-s001.pdf]

Supplementary materials

Figure S1

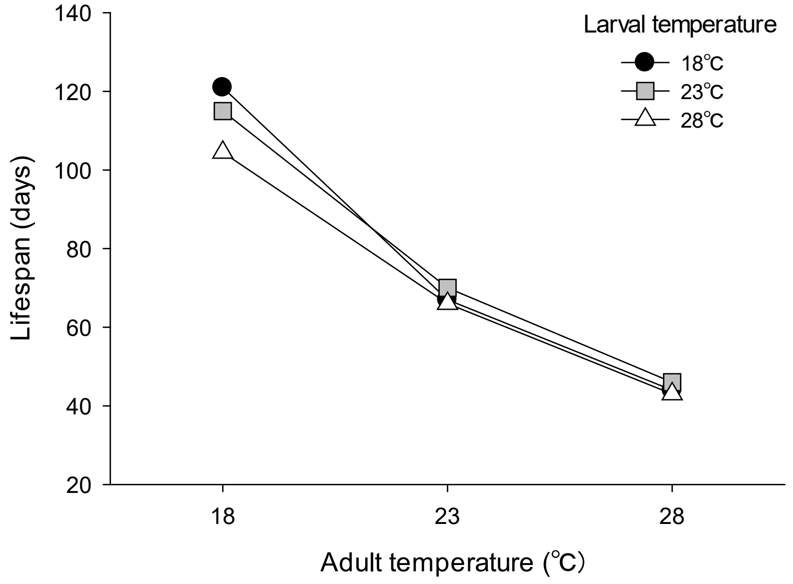

Figure S1. Effect of larval and adult temperature on adult lifespan for female *D. melanogaster*. The values are median.

Figure S2

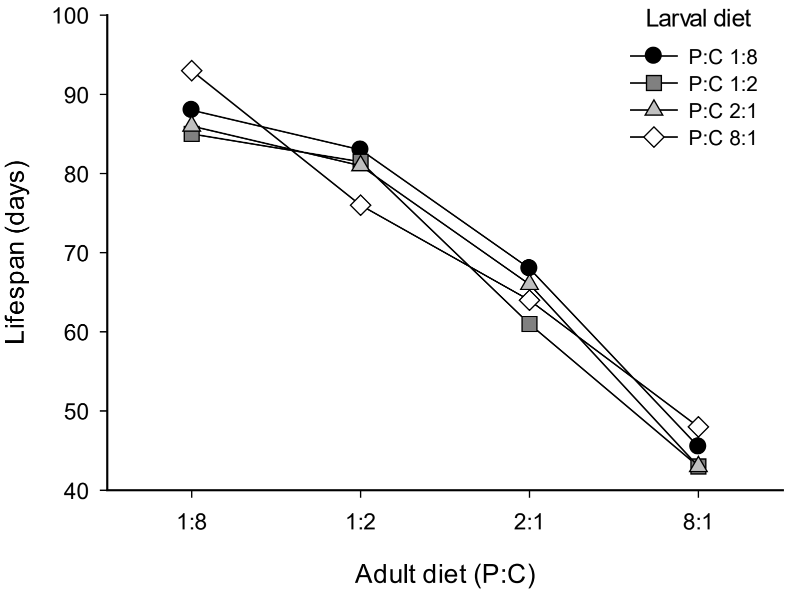

Figure S2. Effect of larval and adult diet on lifespan for female *D. melanogaster*. The values are median.
